# Arsenic trioxide and angiotensin II have inhibitory effects on HERG protein expression: Evidence for the role of PML SUMOylation

**DOI:** 10.18632/oncotarget.17563

**Published:** 2017-05-02

**Authors:** Yu Liu, Duo Li, Dan Nie, Shang-Kun Liu, Fang Qiu, Mei-Tong Liu, Yuan-Yuan Li, Jia-Xin Wang, Yan-Xin Liu, Chang-Jiang Dong, Di Wu, Wei Tian, Jia Yang, Wei Mu, Jia-Tong Li, Dan Zhao, Xiao-Feng Wang, Wen-Feng Chu, Bao-Feng Yang

**Affiliations:** ^1^ Department of Pharmacology, The State-Province Key Laboratories of Biomedicine Pharmaceutics of China, Key Laboratory of Cardiovascular Research, Ministry of Education, College of Pharmacy, Harbin Medical University at Harbin, Heilongjiang 150081, P. R. China; ^2^ Department of Clinical Pharmacy, Key Laboratories of Education Ministry for Myocardial Ischemia Mechanism and Treatment, The 2nd Affiliated Hospital, Harbin Medical University at Harbin, Heilongjiang 150081, P. R. China; ^3^ Department of Oral and Maxillofacial Surgery, The 2nd Affiliated Hospital, Harbin Medical University at Harbin, Heilongjiang 150081, P. R. China

**Keywords:** cardiotoxicity, human ether-a-go-go-related gene, transforming growth factor β1, PML nuclear body, SUMOylation

## Abstract

The human ether-a-go-go-related gene (HERG) channel is a novel target for the treatment of drug-induced long QT syndrome, which causes lethal cardiotoxicity. This study is designed to explore the possible role of PML SUMOylation and its associated nuclear bodies (NBs) in the regulation of HERG protein expression. Both arsenic trioxide (ATO) and angiotensin II (Ang II) were able to significantly reduce HERG protein expression, while also increasing PML SUMOylation and accelerating the formation of PML-NBs. Pre-exposure of cardiomyocytes to a SUMOylation chemical inhibitor, ginkgolic acid, or the silencing of UBC9 suppressed PML SUMOylation, subsequently preventing the downregulation of HERG induced by ATO or Ang II. Conversely, knockdown of RNF4 led to a remarkable increase in PML SUMOylation and the function of PML-NBs, further promoting ATO- or Ang II-induced HERG protein downregulation. Mechanistically, an increase in PML SUMOylation by ATO or Ang II dramatically enhanced the formation of PML and Pin1 complexes in PML-NBs, leading to the upregulation of TGF-β1 protein, eventually inhibiting HERG expression through activation of protein kinase A. The present work uncovered a novel molecular mechanism underlying HERG protein expression and indicated that PML SUMOylation is a critical step in the development of drug-acquired arrhythmia.

## INTRODUCTION

Human ether-a-go-go-related gene (HERG) encodes the rapid component of the delayed rectifier potassium current (IKr) that is the major contributor in depolarizing cardiac action potentials [1]. Moderate inhibition of HERG potassium channels with class III antiarrhythmic agents is beneficial in the pharmacological management of arrhythmias. However, excessive blockade of HERG currents resulting from inherited mutations or adverse drug effects can lead to cardiotoxicity, including sudden death and torsade de pointes, a form of fatal arrhythmia. These undesirable side effects have prompted withdrawal of blockbuster drugs from clinical practice [2]. Because HERG is a significant molecular target of a diverse group of existing cardiac and non-cardiac drugs that frequently cause life-threatening ventricular arrhythmias in clinical settings, the role of HERG in cardiac arrhythmias and drug action has received considerable attention [3]. The gating characteristics, structure-function relationships, cell surface trafficking, and allosteric modulation of HERG have been studied intensively [3, 4].

Drug-induced long QT syndrome is closely related to HERG current inhibition, which can be caused by a diverse group of drugs that either directly block the HERG channel or disrupt HERG channel trafficking to the cell surface [5]. HERG current can be indirectly reduced by a decrease in HERG protein abundance and frequently occurs during drug-acquired arrhythmias. Arsenic trioxide (ATO) is extensively used in the treatment of acute promyelocytic leukemia and solid tumors in clinical practice. The therapeutic use of ATO is burdened by broad cardiotoxicity that is characterized by a prolonged QT interval, which can result in life-threatening torsade de pointes and cardiac death. Substantial evidence has been collected demonstrating that ATO can disrupt the HERG current by interfering with trafficking to the cellular membrane, as well as through reduction in HERG protein abundance [6]. A previous analysis of ATO-induced long QT syndrome revealed that ATO treatment of guinea pigs caused an increase in transforming growth factor β1 (TGF-β1) secretion, while HERG protein expression was decreased [7]. The levels of angiotensin (Ang II) have been found to increase during arrhythmia in cases of cardiac hypertrophy and heart failure. Previous evidence has suggested that Ang II has an inhibitory effect on IKr/HERG currents via AT1 receptors linked to the PKC pathway in ventricular myocytes [8]. Cai et al. also showed that Ang II reduced the expression of mature HERG channel protein by accelerating channel proteasomal degradation through AT1 receptor by PKC activation in HEK-293 cells with stably expressed HERG channel proteins [9]. However, the mechanisms that underlie HERG protein expression in response to these different factors have yet to be elucidated.

Promyelocytic leukemia (PML) has been identified to be a direct target of ATO, which is associated with the pathogenesis of leukemia and solid tumors. PML is the building block for PML-nuclear bodies (PML-NBs) and serves as a protein scaffold in recruiting interaction partners that dynamically shuttle in and out of the multi-functional structures. PML modification by the small ubiquitin-like modifier (SUMO) is essential in recruitment to these nuclear matrix-related structures, and this recruitment is mediated by a series of enzymatic reactions. In the first step of the reaction, the SUMO precursor is activated by SUMO-activating enzyme (E1) in an ATP-dependent manner. Next, SUMO is transferred to the SUMO-conjugating enzyme (E2), UBC9. The E3 SUMO ligase alone is not sufficient for SUMO conjugation to various substrates, but does facilitate SUMO modification. Subsequently, RNF4, the SUMO-targeted ubiquitin ligase (characterized as poly-SUMO-2/3-binding factor) triggers the degradation of poly-SUMO-chain-conjugated substrates through the ubiquitin system [10]. Comprehensive studies of the physiological effects of PML and associated PML-NBs have already been conducted in growth control, DNA repair, and apoptosis [11]. However, the molecular and biochemical mechanisms for ion channel regulation that are associated with PML remain unclear.

This study was performed to elucidate the functions of PML SUMOylation and the associated NBs on HERG protein expression, as well as related regulatory cues. This may facilitate an understanding and potential control of HERG-induced cardiotoxicity, allowing for treatment and prevention in clinical practice.

## RESULTS

### ATO or Ang II inhibited HERG protein expression in cardiomyocytes

**Figure 1:**
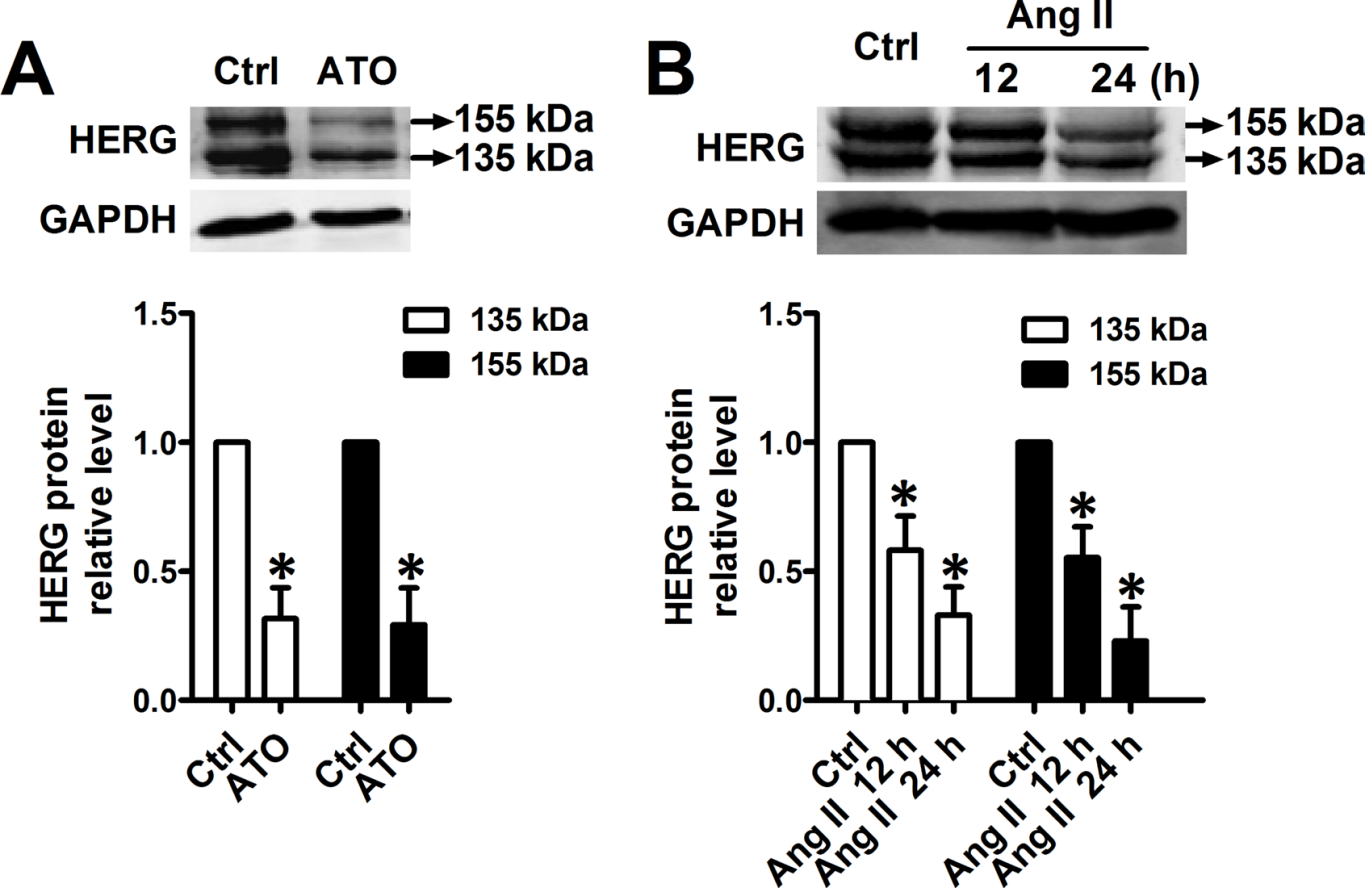
Downregulation of HERG protein expression by ATO and Ang II Immunoblot analysis of HERG protein expression in NMCMs pretreated with (**A**) ATO (2 μM) for 24 h and (**B**) Ang II (100 nM) for 12 or 24 h. Quantitative densitometric analyses of the HERG protein at 135 kDa or 155 kDa are shown in the lower panel. The data represent the mean ± SEM normalized with control (Ctrl) of three separate experiments. *P < 0.05 versus control group.

HERG potassium channels conduct the rapid delayed rectifier K current, which is responsible for cardiac excitability and maintenance of normal cardiac rhythm. HERG is highly expressed in the cell membranes of neonatal mouse cardiomyocytes (NMCMs), where the function of HERG is well understood [12]. Immunoblot analysis indicated that, in cultured NMCMs, HERG protein appears as two bands, a precursor core-glycosylated, immature isoform (135 kDa) and a higher molecular band (155 kDa) which corresponds to the fully-glycosylated, mature HERG protein as described previously [3]. Exposure to ATO for 24 h at a clinically relevant concentration (2 μM) visibly decreased the intensity both of fully-glycosylated and core-glycosylated HERG proteins (Figure 1A). Similarly, 100 nM Ang II also caused a significant decrease in HERG protein abundance in a time-dependent manner (Figure 1B). In addition, exposure of ATO at 2 μM or Ang II at 100 nM did not change cardiomyocyte viability (Supplementary Figure 1).

### Both ATO and Ang II promoted PML SUMOylation and PML-NBs accumulation

**Figure 2:**
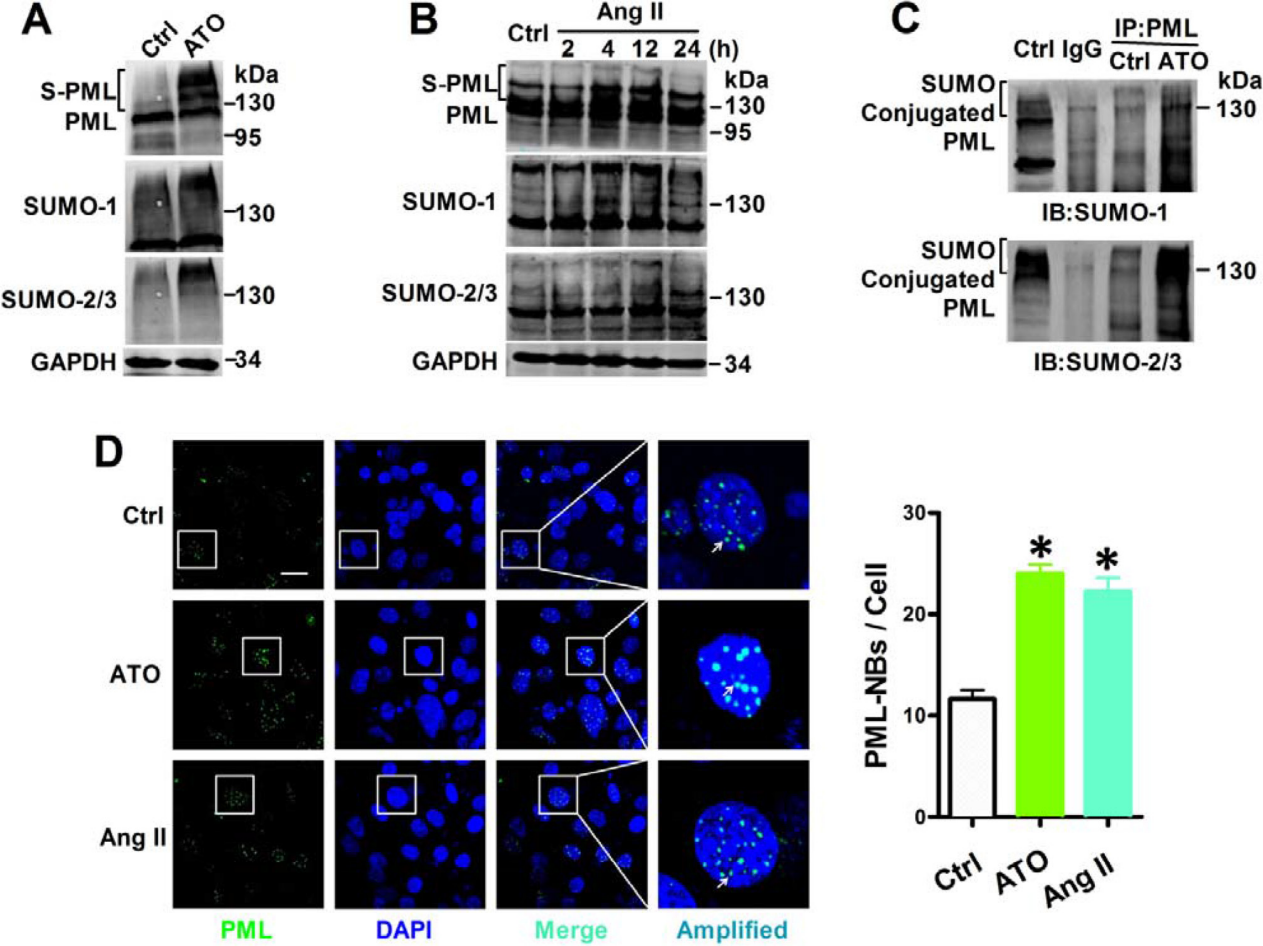
Effects of ATO and Ang II on PML SUMOylation and PML-NBs formation Whole cell extract were treated with anti-PML, anti-SUMO-1 and anti-SUMO-2/3 antibody in NMCMs that were exposed to (**A**) ATO (2 μM) for 2 h and (**B**) Ang II (100 nM) for the indicated periods. The protein molecular masses are in kDa and shown to the right of each panel. The high molecular-mass-weight bands (> 130 kDa) in parentheses represent SUMOylated PML (S-PML). (**C**) Total lysates from NMCMs were immunoprecipitated with anti-PML antibody. The immunopellets then were detected with either anti-SUMO-1 or anti-SUMO-2/3 antibody. (**D**) Confocal immunofluorescent analysis of PML-NBs (green) and nuclei (blue) in NMCMs treated with ATO (2 μM) for 2 h or Ang II (100 nM) for 4 h. Scale bar: 20 μm. Enlarged view of a single PML-NB in the boxed region is shown at higher magnification in the right panel. White arrowheads indicate the typical PML-NBs. *P < 0.05 versus control group. The data shown in Figure A–D are representative of three separate experiments.

In order to further study the level of PML SUMOylation, PML, SUMO-1, and SUMO-2/3 were labeled with specific antibodies in NMCMs. Short-term exposure to ATO induced a dramatic shift towards the SUMO-reactive PML species. The same membranes were probed with anti-SUMO-1 and anti-SUMO-2/3 antibodies, and revealing an increased global expression of SUMOs after ATO stimulation (Figure 2A). Similarly, Ang II triggers a massive shift toward SUMO conjugation PML after exposure for 4 h, while long exposure of Ang II for 12 or 24 h reduced SUMOylated PML levels, which was accompanied by corresponding increased SUMO expression (Figure 2B). Co-immunoprecipitation indicated that the high-molecular-weight PML species (> 130 kDa) was the conjugation of SUMO-1 or SUMO-2/3. Exposure with ATO for 2 h dramatically increased the conjugation of SUMO to PML (Figure 2C).

The multi-functional large multi-protein called PML-NBs, also known as PML oncogenic domains (POD), nuclear domain 10 (ND10), and Kremer bodies—are highly dynamic structures that exist within the nuclei of most mammalian cells [13]. Their biochemical functions are involved in diverse cellular processes, including apoptosis, DNA damage, and gene transcription. For this reason, PML-NBs were examined in ATO- and Ang II-treated NMCMs via immunofluorescence. PML protein was localized mainly to PML-NBs, with approximately 15 proteins per nucleus under normal growth conditions. This distribution was augmented by the addition of ATO, as previously reported [14]. Similarly, stimulation with Ang II further increased both the number and size of PML-NBs in cultured NMCMs (Figure 2D).

### PML SUMOylation decreased HERG protein expression

**Figure 3:**
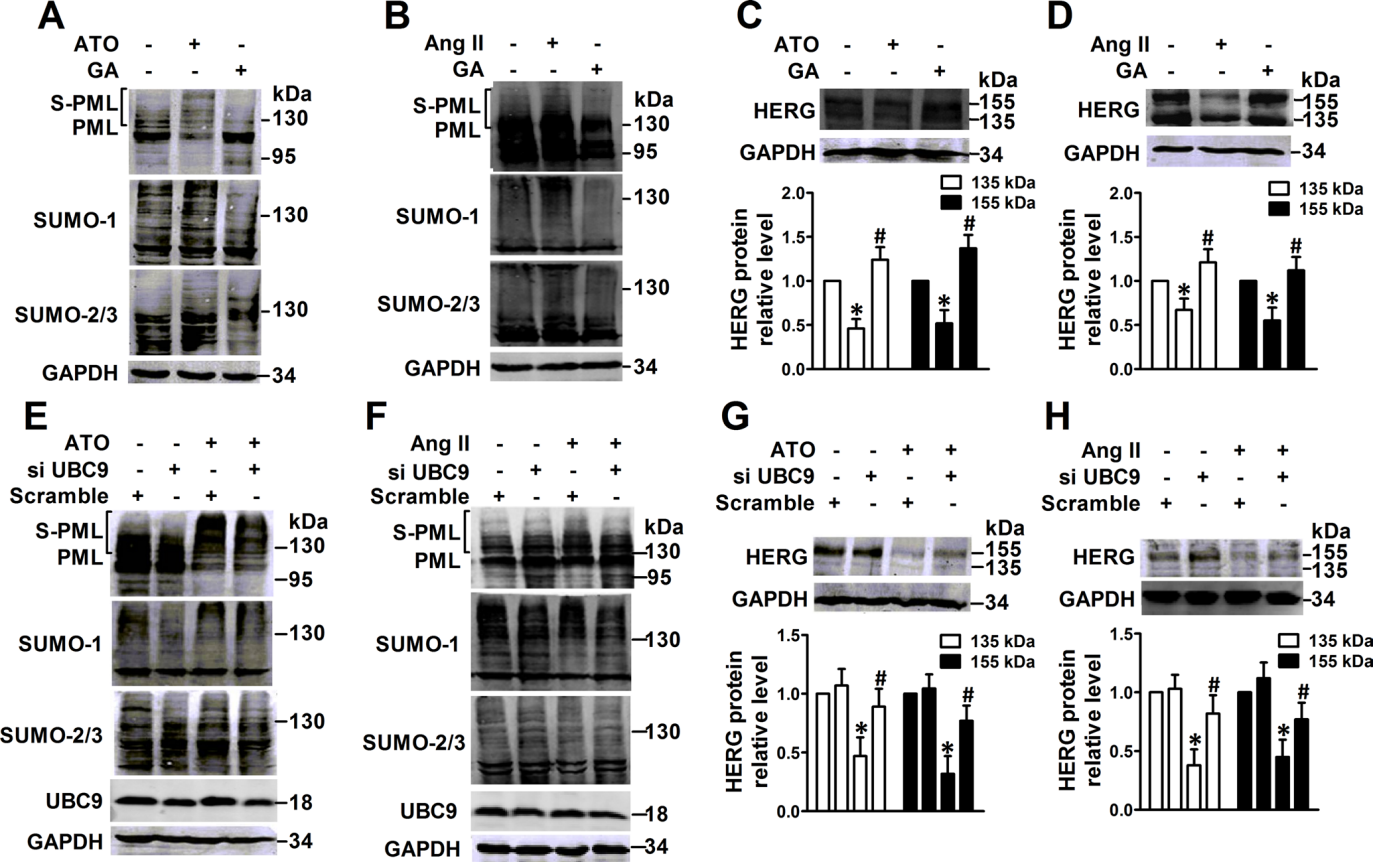
Effect of interference with PML SUMOylation by GA or UBC9 knockdown on HERG expression PML and SUMO molecules were analyzed by immunoblot using whole lysates from NMCMs. The cells were pretreated with GA (50 μM) for 4 h, then treated with (**A**) ATO (2 μM) for 2 h or (**B**) Ang II (100 nM) for 4 h. Immunoblot analysis of HERG protein and quantitative densitometric analysis in the whole lysates from NMCMs. The cells were pretreated with GA (50 μM) for 4 h, then treated with (**C**) ATO (2 μM) or (**D**) Ang II (100 nM) for 24 h. Immunoblot analysis PML, SUMO-1, SUMO-2/3, and UBC9 expression in UBC9-silencing NMCMs treated with (**E**) ATO (2 μM) for 2 h or (**F**) Ang II (100 nM) for 4 h. Immunoblot analysis of HERG expression and quantitative densitometric analysis in NMCMs treated with (**G**) ATO (2 μM) or (**H**) Ang II (100 nM) for 24 h after UBC9 knockdown. The protein molecular mass is given in kDa and shown in the right panel. The high-molecular-weight bands (> 130 kDa) in parentheses indicate SUMOylated PML (S-PML). The data shown in Figures A–H are representative of three separate experiments. The data represent the mean ± SEM normalized with control (Ctrl) of three separate experiments. *P < 0.05 versus control group, ^#^*P* < 0.05 versus scramble + ATO or scramble + Ang II group.

In order to assess the inhibitory effect of PML SUMOylation on HERG protein expression, the SUMOylation chemical inhibitor ginkgolic acid (GA) was used to interfere with PML SUMOylation [15]. Immunoblot analysis indicated that GA treatment partially abolished PML SUMOylation that was induced by ATO (Figure 3A) or Ang II (Figure 3B), consequently reversing the decreased HERG protein expression caused by ATO (Figure 3C) and Ang II (Figure 3D). UBC9, a unique E2-like conjugating enzyme, is essential for conjugation of PML with SUMO [16], and transfection with siRNA against UBC9 significantly decreased the SUMOylated PML and global SUMO expression triggered by ATO (Figure 3E) or Ang II (Figure 3F). NMCMs that showed diminished UBC9 levels were found to have significantly less HERG protein degradation than cells exposed to ATO (Figure 3G) or Ang II (Figure 3H).

**Figure 4:**
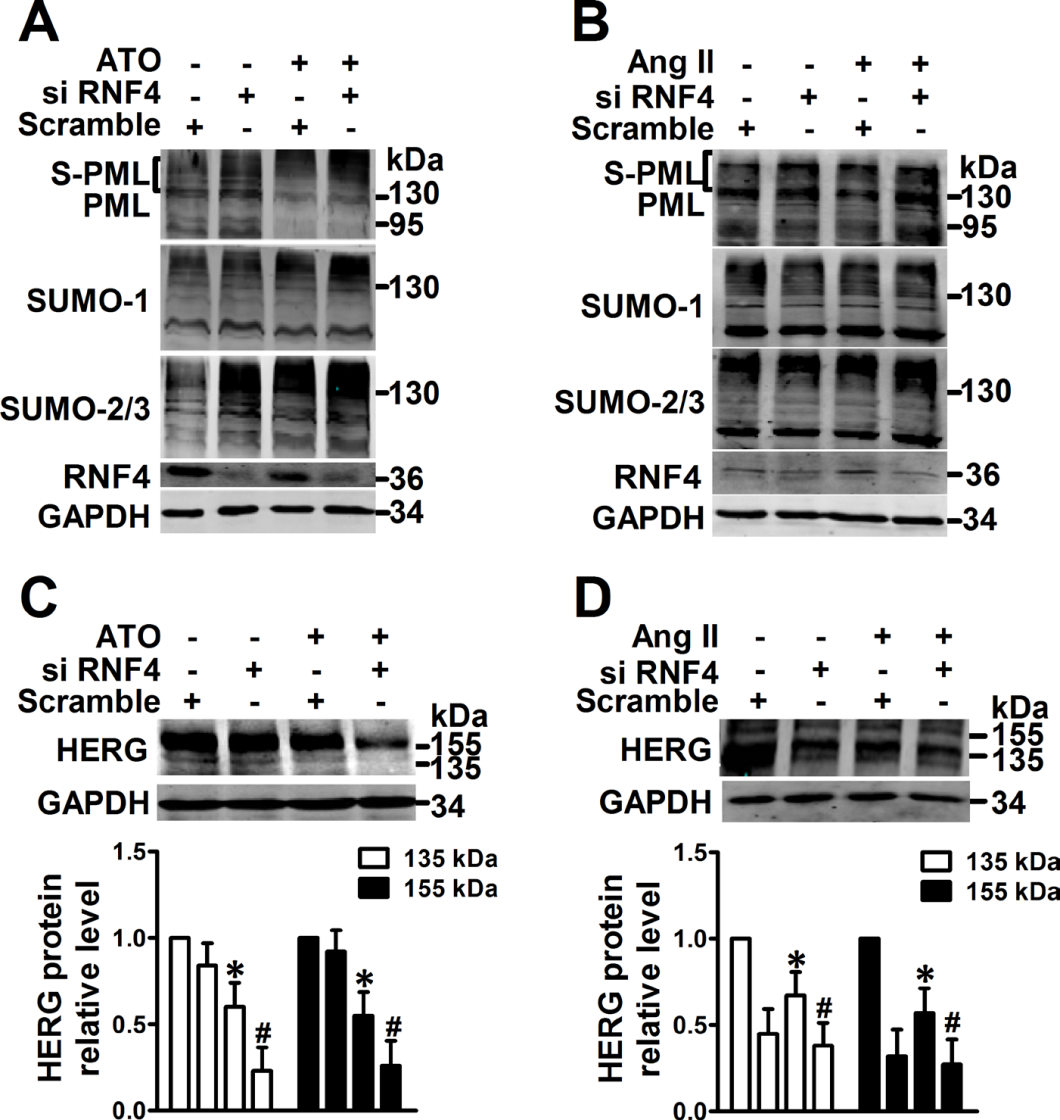
Effect of promotion of PML SUMOylation by RNF4 knockdown on HERG expression PML, SUMO-1, SUMO-2/3, and RNF4 protein were analyzed by immunoblot using the whole lysates from RNF4-silenced NMCMs treated with (**A**) ATO (2 μM) for 2 h or (**B**) Ang II (100 nM) for 4 h. The protein molecular masses are in kDa and shown in the right panel. The high molecular-weight bands (>130 kDa) in parentheses are SUMOylated PML (S-PML). Immunoblot analysis of HERG expression and quantitative densitometric analysis of NMCMs treated with (**C**) ATO (2 μM) or (**D**) Ang II (100 nM) for 24 h after RNF4 knockdown. The data shown in Figure A–D are representative of three separate experiments. The data represent the mean ± SEM normalized with control (Ctrl) of three separate experiments. *P < 0.05 versus control group, ^#^*P* < 0.05 versus scramble + ATO or scramble + Ang II group.

In order to regain PML SUMOylation, RNF4, a RING-finger ubiquitin ligase implicated in arsenic-induced PML degradation via the ubiquitin-proteasome [11], was knocked down in NMCMs. Bands were observed via immunoblotting that were consistent with SUMOylated PML after ATO and Ang II treatment and RNF4 depletion caused a shift in endogenous SUMO conjugates that were consistent with global SUMO expression (Figure 4A, 4B). Less HERG protein expression was noted in NMCMs subjected to RNF4 silencing than in those exposed to ATO (Figure 4C) or Ang II (Figure 4D).

### PML increased expression of TGF-β1 in a Pin1-dependent way

**Figure 5:**
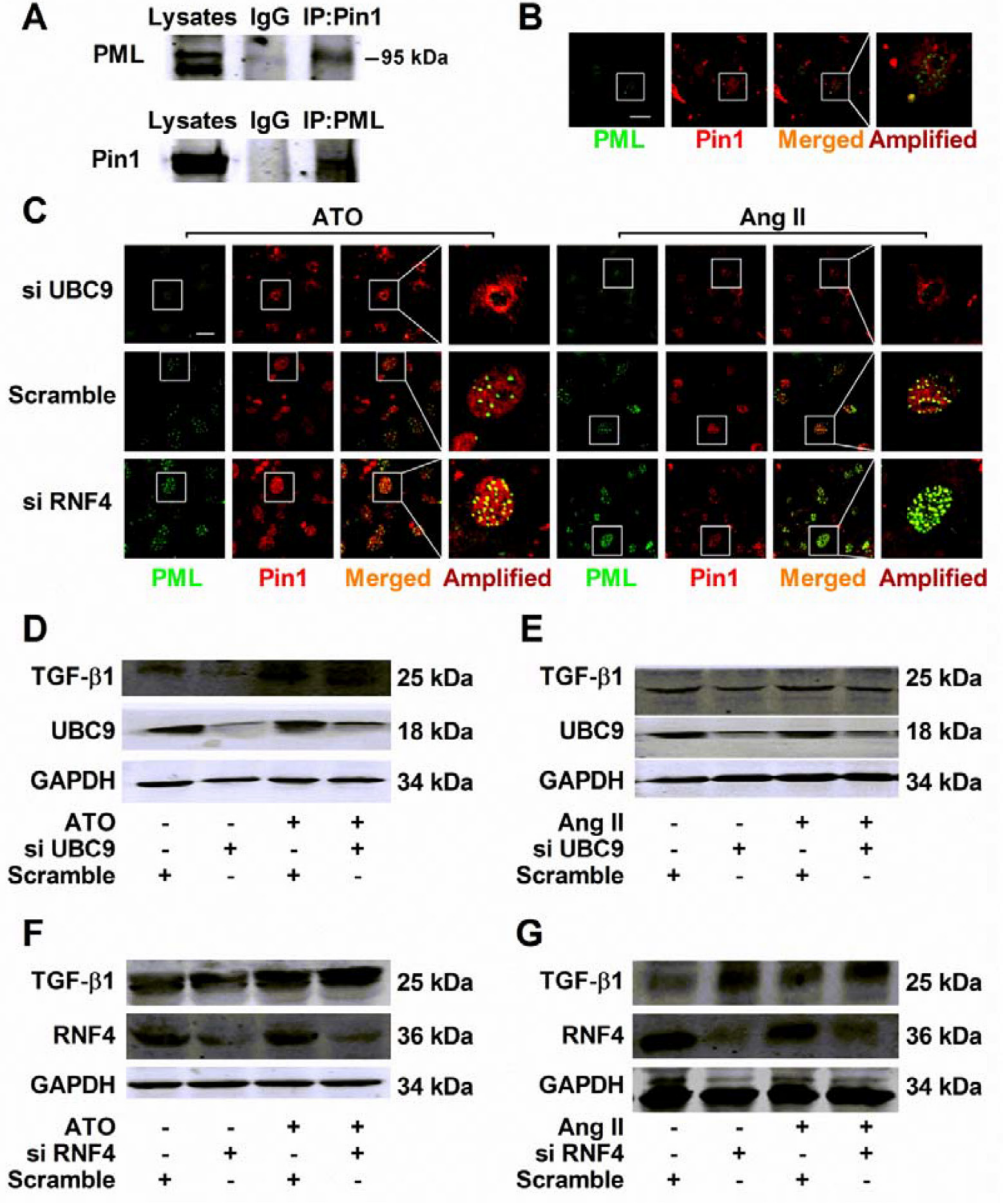
Regulation of TGF-β1 by recruiting Pin1 into PML-NBs during HERG protein degradation (**A**) Co-immunoprecipitation analysis of PML interaction with Pin1 in cultured NMCMs. Total lysates immunoprecipitated with anti-Pin1 and anti-PML antibody were detected with anti-PML and anti-Pin1 antibodies, respectively. (**B**) Representative immunofluorescent staining of NMCMs immunostained with specific antibodies against PML (green) and Pin1 (red). (**C**) Immunofluorescence analysis performed using anti-PML (green) and anti-Pin1 (red) antibodies in NMCMs exposed to ATO (2 μM) for 2 h or Ang II (100 nM) for 4 h and transfected with the indicated siRNAs. Figure B–C: scale bar, 20 μm. An enlarged view of a single PML-NB in the boxed region is shown in the right panel. The representative images of three separate experiments are shown. Immunoblot analysis of TGF-β1 and GAPDH expression in UBC9-silenced NMCMs treated with (**D**) ATO (2 μM) or (**E**) Ang II (100 nM) for 24 h. TGF-β1 and GAPDH expression was analyzed by immunoblot of whole lysates from RNF4-silenced NMCMs treated with (**F**) ATO (2 μM) for or (**G**) Ang II (100 nM) for 24 h. The protein molecular masses are in kDa and are shown in the right panel. The high molecular-weight bands (> 130 kDa) in parentheses are SUMOylated PML (S-PML). The data shown are representative of three separate experiments.

PML-NBs have been implicated in a variety of biological processes via mechanisms regulating the functions, activities, and localization of PML-NBs binding partners, including Pin1 [17]. Importantly, recent evidence indicates that Pin1 controls TGF-β1 mRNA stability in multiple organs [18]. Co-immunoprecipitation studies detecting the PML/Pin1 complex in cultured NMCMs were performed using lysates immunoprecipitated with either anti-PML or anti-Pin1 antibodies (Figure 5A). Double immunofluorescence analysis was conducted to visualize PML-NBs morphology and the interaction with Pin1 under normal conditions (Figure 5B). A small fraction of complexes that contained PML and Pin1 was observed in the presence of ATO or Ang II, while UBC9 knockdown dramatically reduced the number of PML-NBs, as well as sequestering Pin1 into these structures. In RNF4-silenced cells, PML-NBs were augmented with numerous PML foci, leading to a dramatic increase in the co-localization of PML with Pin1 (Figure 5C).

Changes in TGF-β1 levels were assessed after intervention with PML SUMOylation and the suppression of PML SUMOylation by UBC9 knockdown failed to recruit Pin1. This can be attributed to reductions in TGF-β1 upregulation induced by ATO (Figure 5D) or Ang II (Figure 5E). In contrast, RNF4 silencing resulted in TGF-β1 expression that was higher than in NMCMs treated with ATO (Figure 5F) and Ang II (Figure 5G).

### TGF-β1 suppressed HERG protein expression through PKA activation in NMCMs

**Figure 6:**
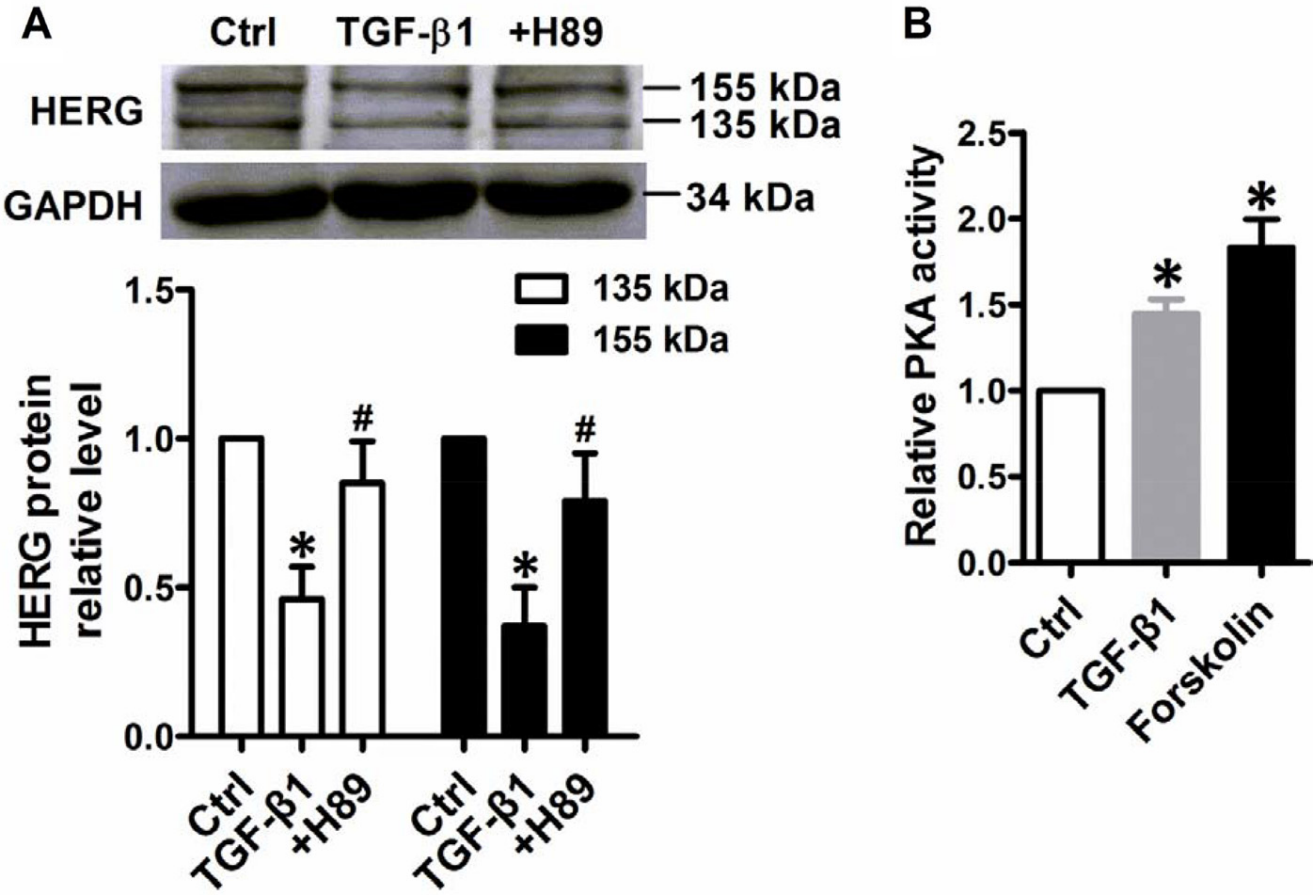
HERG protein expression inhibition by TGF-β1 stimulation dependent of PKA activity (**A**) Immunoblot analysis of HERG protein expression inhibition by TGF-β1 in NMCMs. Cells were treated with TGF-β1 (5 ng/mL) for 24 h or co-treated with H89 (10 mM) and TGF-β1 (5 ng/mL). The results of quantitative densitometric analysis results are shown in the lower panel. (**B**) PKA activity was measured in NMCMs using a kit with TGF-β1 (5 ng/mL) for 24 h or with TGF-β1 and forskolin (10 μM). The data represent the mean ± SEM normalized with GAPDH of three separate experiments. *P < 0.05 versus control group, ^#^*P* < 0.05 versus TGF-β1 group.

Previous research has demonstrated that increases in protein kinase A (PKA) activity significantly inhibited HERG protein expression [7, 19]. Cultured NMCMs were treated with antagonists of PKA-H89 and activators of PKA-forskolin to confirm the role of PKA in TGF-β1-mediated HERG expression. Stimulation with TGF-β1 (5 ng/mL) for 24 h visibly reduced HERG abundance, while downregulation was prevented by treatment with 10 mM PKA antagonist H89 (Figure 6A). TGF-β1 stimulation increased PKA activity and supplementation with 10 μM forskolin yielded similar results (Figure 6B). Together, these observations suggest a role for PKA activity in TGF-β1-suppressed HERG abundance.

## DISCUSSION

**Figure 7:**
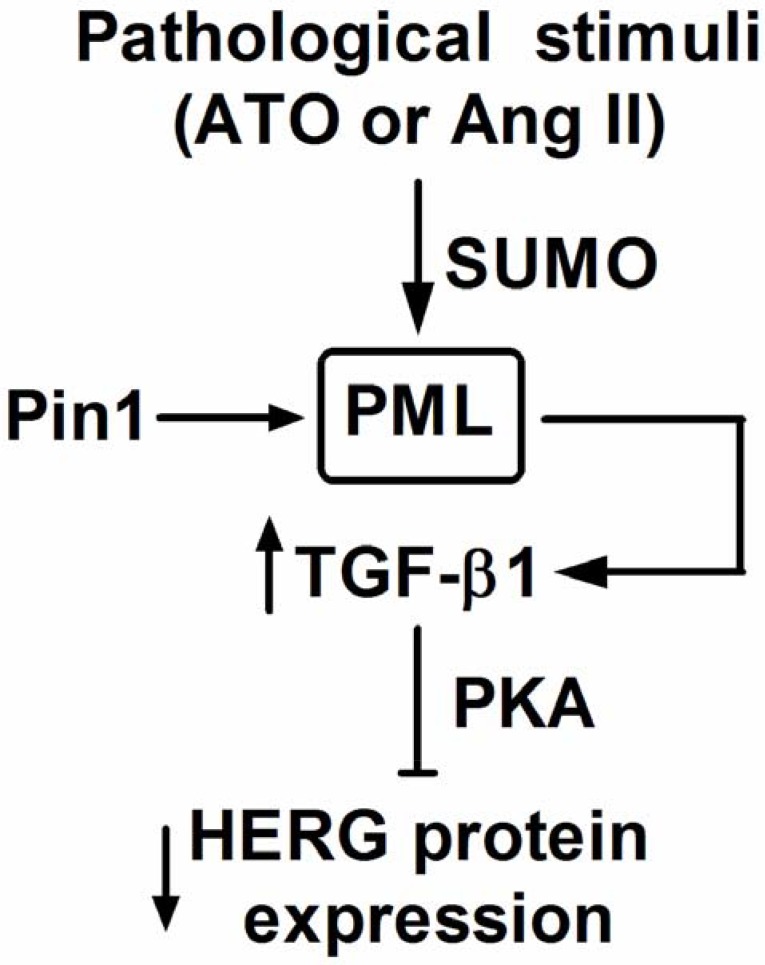
Putative mechanism of PML on HERG protein expression Stimulation of ATO and Ang II increased the SUMO conjugation with PML, as well as NBs formation. PML-NBs promoted the sequestration of Pin1 into PML-NBs and then upregulated TGF-β1 expression, leading to downregulation of HERG via PKA.

In the present study, the effects of PML SUMOylation and associated NBs on HERG protein expression, as well as the mechanisms that underlie its action, were investigated in cultured NMCMs. Primarily, it was found that: (1) exposure of cells to ATO and Ang II significantly reduced the expression of HERG protein; (2) both ATO and Ang II stimulation increased PML SUMOylation and NBs aggregation, promoting recruitment with Pin1; (3) translocation of Pin1 into PML-NBs led to upregulation of TGF-β1, eventually inhibiting HERG expression through PKA activation (Figure 7).

SUMOylation is an enzymatic reaction that includes SUMO activation and requires E1 activating enzyme, SUMO conjugation to the -targeting substrate by UBC9, and modification by several E3 protein ligases. Modulation of these steps, such as by regulatory molecules, controls the SUMO pathway, and Fukuda et al. reported that GA, a small molecular inhibitor, significantly impairs protein SUMOylation by inhibiting formation of the E1-SUMO complex [15]. PML-NBs biogenesis and SUMOylation are also regulated via cooperation between PML, UBC9 and RNF4 [20]. Nacerddine et al. had observed that UBC9-deficient mice had abrogated SUMO pathways and exhibited severe defects in PML-NBs organization, eventually leading to decreased embryonic viability [21]. Significantly, mice deficient in UBC9 fail to form normal PML-NBs and PML remains unmodified by SUMOs [22]. Among SUMO-regulated processes, RNF4 represents a subclass of ubiquitin ligases that target proteins modified by the SUMO for ubiquitin-mediated degradation [23]. PML is characteristic of most substrates degraded in this way. RNF4 targets poly-SUMO-modified PML for degradation through a ubiquitin-proteasome pathway. Michael et al. showed that RNF4 depletion could lead to accumulation of poly-SUMO chains and PML protein [24]. In the present study, RNF4 knockdown further decreased ATO- or Ang- II-induced HERG channel degradation due to PML accumulation, independent of its E3 ligase activity. Guo et al. reported that another E3 ubiquitin ligase, Nedd4-2, ubiquitinates and eliminates mature HERG channels [25]. Cav3 decreases the expression of HERG via recruiting Nedd4-2 from the cytosol to the membrane and prevents its interaction with mature HERG channels [26].

A variety of stresses can alter the entire SUMO conjugation pathway, as well as the morphogenesis and partner recruitment of PML-NBs. These include heavy metals, interferon, and viruses, while arsenic very specifically regulates PML SUMOylation. Herein, the dynamic changes in PML-NBs after Ang II treatment were analogous to previous reports of NBs formation induced by interferon, which boosts PML expression [27]. As both the number and size of PML-NBs were dramatically increased, it is likely that pre-existing PML may be recruited into NBs after Ang II exposure. PML-NBs are composed of a striking variety of associated proteins, and PML-NBs can affect many different cellular functions by activation, inhibition, or localization of interaction partners [17]. Chapman et al. confirmed that ceramide-induced modification of HERG currents and an associated rapid decline in HERG protein can be caused by protein ubiquitinylation and subsequent lysosomal degradation [3]. A more complete understanding of the other molecular components involved in PML-mediated HERG expression is necessary to determine the biological function and biomedical significance of HERG.

The effect of TGF-β1 treatment on regulation of the PKA activity may differ in different cell types. For example, Zhang et al. suggested that TGF-β1 upregulates Kv2.1 channel expression and IKr currents without affecting the activity of PKA in rat primary cerebellar granule neurons [28]. However, a previous study showed that incubation of TGF-β1 for 15 min induces PKA activity in rat mesangial cells and mink lung epithelial cells [29]. Giannouli et al. have demonstrated that TGF-β inhibits the proliferation of fetal skin fibroblasts by the activation of PKA [30]. In our study, we showed that TGF-β1 (5 ng/mL) for 24 h increased PKA activity and significantly reduced HERG abundance in NMCMs.

It has recently been demonstrated that the HERG potassium channel is regulated by PKA in Xenopus oocytes, in a CHO cell line, and in isolated guinea-pig ventricular myocytes. Activation of the β-adrenergic system and subsequent elevation of the intracellular concentration of the second messenger, cAMP regulates HERG channels. Previous studies have suggested that adrenergic signal transduction exerts distinct inhibitory effects on cardiac HERG/IKr currents either through PKA-mediated phosphorylation of the channel protein or via direct interaction with the cAMP binding site of HERG [30, 31]. Shu et al. demonstrated that exposure of cells to PKA activators for 10 h significantly impaired HERG K^+^ channel function and reduced HERG K^+^ current density via generation of ROS [19, 31]. Controversially, Chen et al. also demonstrated that isoproterenol administration increased HERG abundance in HEK-293 cells co-transfected with HERG and β-adrenergic receptor. This study also demonstrated that elevated PKA activity via forskolin at 10 μM produced a sustained increase in the abundance of HERG channel protein in stably transfected HEK-293 cells [32]. Another study explained this by showing that TGF-β stimulates PKA activity independent the intracellular cAMP levels in murine mesangial cells [29]. β-adrenergic receptor or forskolin, an adenylyl cyclase activator, both increase PKA activity through the elevation of intracellular levels of cAMP. Thus, activation of PKA by different signals may have pleiotropy effects on HERG channel expression. It can be concluded that TGF-β reduced HERG channel expression through PKA activation, possibly independent of the cAMP levels.

PML proteins have received much attention due to their pluripotent biochemical and physiological involvements in cancer, the immune system, and inflammatory conditions. However, the function of PML in cardiovascular disorders has only recently begun to be elucidated. This is the first study to identify an effect of PML SUMOylation on the HERG protein and provides a basis for the regulatory roles of PML SUMOylation in HERG expression, as well as a potential strategy for treating and preventing HERG-associated cardiotoxicity.

## MATERIALS AND METHODS

### Primary culture of neonatal mouse cardiomyocytes (NMCMs)

This investigation was approved by the Animal Experiments Committee of the Harbin Medical University and conformed with the Guide for the Care and Use of Laboratory Animals published by the United States National Institutes of Health (NIH Publication No. 85-23, revised 1985). NMCMs were obtained from 1–2-day-old Kunming neonatal mice as described previously. In detail, the ventricle tissues of the neonatal mice were finely minced and subjected to enzymatic digestion with 0.25% trypsin (Beyotime Biotechnology; China) and mechanical dissociation. Suspensions were harvested and seeded into 6-well culture plates. Non-myocytes were discarded based on different adhesion time between cell types, leaving NMCMs attached at a final density of 10^6^ cells/well in DMEM containing 10% fetal bovine serum (HyClone, Logan, UT, U.S.), 100 U/mL penicillin, and 100 mg/mL streptomycin (Beyotime Biotechnology; China). BrdU (5-bromo-20-deoxyuridine) was added into the culture medium to prevent the growth of non-myocytes.

### Cell transfection and treatment

siRNA silencing and cell treatments were performed as described previously. After 72 h of incubation, the NMCM medium was replaced with serum-free DMEM overnight before treatment with different reagents. Angiotensin II (Ang II) (Sigma-Aldrich; St Louis, MO, U.S.) was supplemented at 100 nM and ATO (Harbin YI-DA Pharmaceutical Company; China) was supplemented at 2 μM for the indicated times. siRNA was purchased from GenePharma (Shanghai, China) with the following sequences (Table 1).

**Table 1:** siRNA sequences

Gene		Sequence
UBC9	sence	5′-CAAUGAACCUGAUGAACUG-3′
	antisence	5′-CAGUUCAUCAGGUUCAUUG-3′
RNF4	sence	5′-GGAAACUGUUGGAGAUGAA-3′
	antisence	5′-UUCAUCUCCAACAGUUUCC-3′
Scramble	sence	5′-UUCUCCGAACGUGUCACGU-3′
	antisence	5′-ACGUGACACGUUCGGAGAA-3′

All the genes correspond to *mus musculus*.

Transfections were performed using X-treme GENE siRNA transfection reagent (Roche, Indianapolis, IN, U.S.) in accordance with the manufacturer's protocol. Samples were analyzed after 36 or 48 h of transfection and all results were compared to transfections with a non-targeting siRNA. NMCMs were washed once with sterile PBS and then starved with serum-free medium for 4–6 h. The siRNA and X-treme were separately diluted with 250 μL of Opti-MEM reduced serum medium (Gibco, Grand Island, NY, U.S.) for 5 min, then mixed together and incubated for 20 min. The mixture was added to cells and cultured at 37°C. After incubation for 4–6 h, fresh culture medium supplemented with 10% fetal bovine serum was replaced with fresh culture medium and the cells were cultured at 37°C for further treatment.

### MTT assay

Cell survival was examined by the 3-[4,5-dimethylthylthiazol-2-yl]-2,5 diphenyltetrazolium bromide (MTT) assay (Sigma, St. Louis, MO, U.S.). Cells were seeded onto 96-well flask. At specific points in time, MTT was added to each well, and the cells were then incubated at 37°C for 4 h. DMSO was then added to each well. A microplate reader was used to measure the optical density (OD) value at 490 nm after shaking for 10 min.

### Immunoblot analysis

Total protein was extracted from cells as previously described. Cells were lysed in RIPA buffer (Beyotime Biotechnology; China) containing 1% protease inhibitors (Roche Diagnostics, Mannheim, Germany) or 20 mM N-ethylmaleimide as appropriate (Sigma-Aldrich; St. Louis, MO, U.S.). The lysates were separated on an 8% or 12% SDS-PAGE gel and then transferred to nitrocellulose filter membranes. After being blocked in 5% non-fat milk, the membranes were incubated with the following primary antibodies: anti-PML (1:500 dilution; MBL, Nagoya, Japan), SUMO-1 (1:200 dilution; Santa Cruz Biotechnology, Santa Cruz, CA, U.S.), SUMO-2/3 (1:500 dilution; Abcam, Cambridge, U.K.), UBC9 (1:500 dilution; Cell Signaling Technology, Beverly, MA, U.S.); RNF4 (1:500 dilution; Abcam, Cambridge, U.K.); anti-TGF-β1 (1:500 dilution; Cell Signaling Technology, Beverly, MA, U.S.) and anti-HERG (1:500 dilution; Alomone labs, Jerusalem, Israel). GAPDH (1:10,000 dilution; Research Diagnostics, Concord, MA, U.S.). Goat anti-rabbit (1:10,000 dilution; Alexa Fluor 700 conjugated; Molecular Probes/Life Technologies) served as the secondary antibody. Immunoblots were imaged using an LI-CORE Imaging System (LI-COR Biosciences, Lincoln, NE, U.S.) and Odyssey software was used for quantification of bands normalized to GAPDH.

### Co-immunoprecipitation

Cells were lysed in RIPA buffer (Beyotime Biotechnology; China) and 500 μg of protein was quantified after concentration measurement using a BCA kit (Beyotime Biotechnology; China). Primary antibodies against PML (Santa Cruz Biotechnology, Santa Cruz, CA, U.S.) and Pin1 (Santa Cruz Biotechnology, Santa Cruz, CA, U.S.) were incubated with the protein samples at 4°C overnight on a shaker. Protein A/G agarose beads were added to the samples and allowed to incubate at 4°C for 8 h. After centrifugation at 3,000 × g for 5 min, the beads were gently collected and rinsed five times with TBST. The samples were denatured in loading buffer and the purified proteins were then analyzed using antibodies specific for PML (Santa Cruz Biotechnology, Santa Cruz, CA, U.S.), SUMO-1 (Santa Cruz Biotechnology, Santa Cruz, CA, U.S.), SUMO-2/3 (Abcam, Cambridge, U.K.) or Pin1 (Santa Cruz Biotechnology, Santa Cruz, CA, U.S.) by SDS-PAGE.

### Immunofluorescence

Cells were plated on coverslips, washed in PBS, and fixed with 4% paraformaldehyde for 30 min at room temperature. After washing with PBS, cells were permeabilized for 1 h with 0.1% Triton X-100 in PBS. Next, cells were washed with PBS and blocked in 30% normal goat serum for 1 h at 37°C. Cells were incubated overnight with antibodies against mouse PML (1:100; MBL, Nagoya, Japan) or Pin1 (1:100; Thermo Scientific, Hudson, NH, U.S.). After three washes with PBS, cells were stained with FITC-488- or Alexa-595-conjugated secondary antibodies (1:500; Invitrogen, Carlsbad, CA, U.S.) for 1 h at room temperature. Finally, cells were stained with DAPI (1:50; Beyotime Biotechnology; China) and fluorescence signals were detected using the 60× oil objective on a confocal fluorescence microscope (Olympus, Hamburg, Germany). Images were obtained and processed using the Image-Pro Plus6.0 software program. PML was quantified by counting 100 cells per sample, and experiments were performed in triplicate.

### Protein kinase A (PKA) activity assay

PKA activity was assessed using a ProFluor PKA Assay, as described previously (Promega, Madison, WI, U.S.) [7]. Briefly, NMCMs were treated with either TGF-β1 (5 ng/ml) or forskolin (10 μM) for 24 h. The cells were washed then with PBS and lysed in ice-cold lysis buffer. Equal amounts of the quantified lysates were mixed with the reaction mixture. Then the kinase dilution solution, diluted PKA, and ATP solution were added to each well. The mixture was incubated at room temperature for 20 min, followed by adding protease solution and incubation for another 30 min. Finally, stabilizer solution was added to each sample. Data was collected using an excitation wavelength of 485 nm and an emission wavelength of 530 nm.

### Statistical analysis

Statistical analysis was performed with Prism 5 (GraphPad Software, Inc.). Data are expressed as mean ± SEM. Significant differences between mean values were evaluated with the Student's *t*-test for comparison between 2 groups. Multiple comparisons were performed using one-way ANOVA and Tukey's post hoc tests. For more than 2 groups, 2-way ANOVA was performed. A *P* value < 0.05 was considered to be statistically significant.

## SUPPLEMENTARY MATERIALS FIGURES


