# Supplementary material for: Arsenic trioxide and angiotensin II have inhibitory effects on HERG protein expression: Evidence for the role of PML SUMOylation
Source: Oncotarget. 2017 May 2;8(28):45447–58. doi: 10.18632/oncotarget.17563 (PMC5542199; doi:10.18632/oncotarget.17563)
Supplement: Supplementary file 1 [file oncotarget-08-45447-s001.pdf]

Arsenic trioxide and angiotensin II have inhibitory effects on HERG protein expression: Evidence for the role of PML SUMOylation

Supplementary Materials

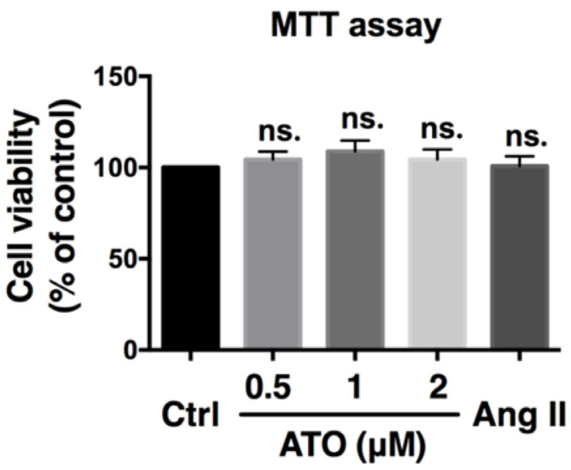

Supplementary Figure 1: The MTT result of NMCMs after treatment with ATO or Ang II. Exposure to low concentrations of ATO (0.5, 1, 2 μM) or Ang II (100 nM) for 24 h showed no effect on cardiomyocyte viability as measured by the MTT assay. ns: no significance.
